# Monthly Summary

**Published:** 1900-03

**Authors:** 


### Monthly Summary.

THE BRUNETTE PEOPLES OF EUROPE.—Sergi has given, in "Science Progress," the results of his study of the brunette races and their migrations to the Mediterranean belt. To his mind the origin of those races is a question of racial rather than linguistic migration. While admitting that Aryan speech among modern and historic peoples throws the burden of proof on any one who essays to find an origin other than Aryan for those peoples, Sergi makes bold to assume that the race or races with which Aryan speech originated may have been at all times few in numbers, and may further have been long since extinct; and consequently that all the Aryan-speaking races of historic times may have, at one time or another learned Aryan speech, without acquiring more than a slight tincture of Aryan blood. The ground is thus left open for an examination of the question from a point of view primarily anthropologic, and based in the first place on physical, viz., morphologic, criteria of natural kinship between the races to be examined. A survey of the whole Mediterranean shore line has led him to the conclusion that its earliest recognizable inhabitants and their descendants, who form the great mass of the present population, belong to a single closely connected group of races; that their earlier home is to be looked for in the former fertile interior of northern Africa, and not improbably in or near the upper valley of the Nile; and that the peninsulas of South Europa and Asia Minor have been peopled thence along several distinct routes which mainly follow the course of the pliocene land-bridges, or former land connections. The "Mediterranean race," thus described, has the following characteristics common to all its branches: The outer complexion is typically brown; brown skin, brown eyes, brown hair, abundant and always more or less wavy. It is thus equally distinct from the blonde white races which bound it on the north, and from the negro races of Africa. Modifications of the brown tint are found in all branches of the race, but are conceived to be due to intermixture either with the earlier aborigines or with subsequent intruders. The body is well-proportioned, the face oval, the nose rather narrow, the orbits wide and set horizontally, the forehead high and nearly vertic, the cheek-bones neither wide nor very high, the face not flattened, but if anything a little prominent in front; the neck long and well-rounded, and the features mobile and expressive. It is, in fact, the familiar brunette type which every one recognizes who has traveled to any extent along the Mediterranean. Determined by certain types of skull, the "Mediterranean race" appears, wherever it is found, as a collocation, more or less uniformly complete, of a number of such related types; and from this it is inferred that the race was already composite in the farthest area of origin to which it can be traced. This centre is placed by Sergi in the upper valley of the Nile, on the ground that here, among the Abyssinians, Gallas and Somalis, the characteristic collocations of types are most completely exhibited; the dusky complexion of a large proportion of these races at the present day being discounted, partly by their long-continued exposure to a more tropic climate than any other branch of the race, and partly by the certainty of continuous infusion of a negroid strain from the south.—"Popular Science News."

---

COLLEGE ATHLETICS.—A conspicuous feature of education in this country, one of its side lines, so to speak, is that of athletic games for the physical development and amusement of the students. Every college and school, of high and low degree, has its gymnasium for physical training and its athletic teams of trained men among the students, to play foot ball, base ball, and other games which require a high degree of skill and physical endurance. Even the girls' schools have their athletic games also nowadays. This brings to the front students of rare physical powers, and makes them the champions of their fellows in competitive games with other similar institutions, and thereby develops and fosters college pride. This is a commendable spirit, for pride in and admiration for their champions sinks personal selfishness and unites the students by a common bond of fraternity and good feeling. It stimulates an *esprit de corps*, as perhaps nothing else could do. The exuberance of animal spirits in the young makes them desire to engage in contests, in which physical strength and skill are put to the test. This spirit comes down to us from classic times, when games of strength and skill were so much cultivated and applauded. It is a wholesome spirit, and should be encouraged by all institutions of learning, where the young are congregated together.

College athletics also develop a pride in the institution with which the student is connected. He is drawn closer to his *alma mater*, and his ardent desire to see her sons victorious in contests with other institutions stirs his soul and creates a vigorous patriotism for her and her champions. His feelings and affections are aroused, and he becomes enthusiastic in his devotion to her interests in general as well as wishing to see her sons win in the contests in the athletic field. He rejoices when his college team is victorious, and is sorrowful when they are defeated, and even in after years it "warms the cockles of his heart" to hear that his successors at his *alma mater* are maintaining her prestige by winning victories on the gridiron or the diamond. It is a bond between him and his college that never grows old, nor fades.

To the individual student, the pursuit of athletic games and exercises is valuable, because of the development of a better physical organization and strength, and laying up stores of energy for the future. The English appreciate this better than we, when they encourage games and sports that develop vigorous physical organization in their boys. Wellington said that the battle of Waterloo was won upon the cricket-fields of Eton by the traing that English boys received there. Military men insist upon the value of such games for young men who are to be soldiers, because of their effect in causing a better physical development and vigor. But the argument holds for the other walks of life as well, for in the fierce struggle for existence there is great need of physical strength and endurance, which these exercises will assist materially in developing and laying up stores of for the future, to fortify the body against the needs of even ordinary life.

This exercise also contributes to mental vigor and will tend to produce that most desirable combination, "a sound mind in a sound body." The student who plays well in the athletic field is apt to work well in tbe study. His brain is invigorated by his physical efforts, and his mental faculties are more active, which enables him to pursue and grasp difficult problems with greater ease. In addition to this, college athletics give birth to and cultivate that most wholesome of mental emotions—*Enthusiasm.* Enthusiasm is the tonic of the mind. An enthusiastic interest in something brings sunshine to the soul. It clears the mind, it warms the heart, it drives away despondency and blues, and especially for the student banishes the demon of homesickness, which is such a source of unhappiness to the average student. Such enthusiasm college athletics gives to boys, so this emotion should be encouraged, and besides it takes his thoughts from things that he might pursue for amusement—for the student must be amused—that would not only be useless, but might be harmful and pernicious, and will direct his thoughts into healthful channels.—A. H. Thompson, in *Stomatologist.*

---

UNDERCUT MODELS.—A writer in the November Items of Interest, in an article entitled "Zinc Dies," asks for other methods of casting "undercut" models.

Prepare the model as for any case, always flaring the sides so that it will drop readily from the mould and shellac. Oil as far as the undercut extends, set upon a glass slab or other smooth surface; mix plaster and coarse, short fiber asbestos, equal parts, not too thin, and spread upon the undercut surface about one-quarter inch thick at the base, and half as thick at the top of the model; when hard, trim smooth, and trim the ends at a bevel so it can be easily replaced in the mould, dry thoroughly and mould; it and the model drop out readily, replace the core in the mould and cast the die. The process is simple and effective.

Oiled sand is of great advantage to the dentist who desires to expedite his work, as it is always ready for use, but cannot be used with zinc, as it is poured so hot it burns the oil, but there is no necessity for using zinc, as a proper babbitt metal is far preferable, being the only alloy that has all the requisite qualities for dental dies, and produces the most satisfactory results, as demonstrated by nearly fifty years use, after several years use of zinc.—*Items of Interest.*

IS THE ENAMEL A VITAL TISSUE?—Dr. R. R. Andrews, of Cambridge, with Drs. Williams, Black and others, claims that after the enamel is once formed there is no organic matter in it, and that it has no vitality. Dr. Andrews, in writing upon the subject, says:

"I believe that enamel, fully formed, is nothing more or less than a coat of mail, supplied by nature to protect the dentine, and subserve the processes of mastication, and I doubt if it be possible, in perfectly formed human enamel, to find so much as a trace of organic matter within the substance of the enamel itself, or at its surface. Near its junction with the dentine there are nearly always found slight prolongations of the organic tissue, from the dentine, within its substance, and those are numerous enough at this point to account for the one to three per cent. of organic matter found in enamel on analysis.

"The enamel is lifeless; there is absolutely no difference to be found by the microscope, in the appearance of the enamel of a live tooth, from that of a tooth that has been a long time dead."—International Dental Journal.

---

DEATH FROM CUTTING A WISDOM TOOTH.—M. Heydenreich reported the case of a man, thirty-three years of age, brought to his clinic and said to be suffering from mumps. There was high and persistent fever, rising to 104 degrees F., with agitation, delirium, stiffness of the jaws, and swelling over the right parotid extending into the neck. When M. Heydenreich saw the patient, on the third day of the grave symptoms, the condition seemed to have improved. The temperature was from 102.5 degrees to 100.4 degree, consciousness had returned, and the swelling was strictly limited to the angle of the jaw. The patient could open his mouth, and a drop of pus escaped by the jaw. All the teeth were there. It was certainly a case of suppurative osteitis of the inferior maxilla, due to the eruption of a wisdom tooth. There was not at this time any indication calling for operative measures. The next day, however, the patient became semi-prostrate, and in the evening the temperature rose to 104.9 degrees F.; on the fifth day he was taken in a moribund condition to the hospital.

There was complete left hemiplegia. A free incision was made by means of the thermal cautery as far as the zygoma, but no pus was found. He died next day at mid-day, the temperature being 98.9 degrees F. The autopsy disclosed pus on the right side between the cranial vault and the meninges up to the level of the convexity, towards the median region, and suppurative osteitis of the cranium. On opening the meninges a bed of very thick greenish-yellow pus (showing meningo-encephalitis) was laid bare. There was no lesion in the interior of the brain.—*New York Medical Journal.*

---

AMALGAM FILLINGS IN TEETH.—Mrs. W———, aged about thirty, tall, slender, dark hair and eyes, sallow complexion, generally unhealthy appearance; enlarged indurated submaxillary glands. Some years ago had had an enlarged tumor of some kind taken from right side of neck, back and below the ear; also suffering from indigestion, headache, neuralgia of face and jaws. All symptoms indicated mercury. She had many large amalgam fillings in the teeth and a red rubber plate. I assured her they were the cause of her trouble. After some months' treatment without decesive benefit, she consented to have them removed, and gold fillings and a black rubber plate replaced the former fillings. Then merc-viv., cm., one dose, and S. L. was given. Within three weeks the enlarged glands were visibly diminished, health and complexion much improved. Six weeks later enlargement of glands diminished by half, complexion and health good; the lady well pleased and glands still diminishing at last interview. Within three months' more treatment the enlargement of the glands will be entirely gone—William L. Morgan, M. D., Baltimore, M. D., in "Medical Visitor."

---

A THIEF steals your money behind your back. A dental quack swindles you before your face, and you do not discover it until too late. You can punish the former if you catch him. But the latter has a license to laugh at your folly.—"Dominion Dental Journal."

HOT WATER DRINKING.—There are four classes of persons who should not drink large quantities of hot water. These are as follows :

1. People who have irritability of the heart. Hot water will cause palpitation of the heart in such cases.

2. Persons with dilated stomachs.

3. Persons afflicted with "sour stomach."

4. Persons who have soreness of the stomach, or pain induced by light pressure.

These rules are not for those who take hot water simply to relieve thirst better than cold water, and for that purpose is not to be condemned. But hot water is an excitant, and in cases in which irritation of the stomach exists, should be avoided.—"Indiana Lancet."

---

TO THE EDITOR OF AM. JOURNAL OF DENTAL SCIENCE.

